# Comprehensive evaluation of the liquid fraction during the hydrothermal treatment of rapeseed straw

**DOI:** 10.1186/s13068-016-0552-8

**Published:** 2016-07-13

**Authors:** Zhi-Wen Wang, Ming-Qiang Zhu, Ming-Fei Li, Jun-Qi Wang, Qin Wei, Run-Cang Sun

**Affiliations:** College of Forestry, Northwest A&F University, Yangling, 712100 China; Beijing Key Laboratory of Lignocellulosic Chemistry, Beijing Forestry University, Beijing, 100083 China

**Keywords:** Rapeseed straw, Hydrothermal treatment, Monosaccharide, Oligosaccharide, 2D NMR

## Abstract

**Background:**

The requirement for efficient and green conversion technologies has prompted hydrothermal processing as a promising treatment option for sustainable biorefinery industry. The treatment has been applied to process plenty of lignocellulose materials, yielding abundant high value-degraded products, especially the products in the liquid fraction. Therefore, it is essential to systematically evaluate the degraded products in aqueous fraction by comprehensive analysis and structural characterization during the treatment.

**Results:**

Rapeseed straw was hydrothermally treated at temperature ranging from 145 to 205 °C for various retention time (15, 30, 60 and 120 min), and the degraded polysaccharides and lignin products in aqueous phase were systematically evaluated by comprehensive analysis and structural characterization. Results showed that with an increase of severity, the polymers were gradually depolymerized resulting in a decrease of the molecular weight from 8430 (log *R*_0_ 3.26) to 2130 g/mol (log *R*_0_ 5.08), an increase of oligosaccharides from 19.44 (log *R*_0_ 2.88) to 99.94 g/kg (log *R*_0_ 4.32) and an increase of monosaccharides from 0.91 (log *R*_0_ 2.88) to 30.43 g/kg (log *R*_0_ 4.37). With the increase of monosaccharide degradation components (8.26 to 125.68 g/kg), the saccharides gradually decreased after its maximum value. The maximum yield of oligosaccharides (99.94 g/kg) accompanying a relatively low level of monosaccharides (17.77 g/kg) was obtained at a high temperature (190 °C) for a short reaction time (15 min). The degraded polysaccharides had a linear backbone of (1 → 4)-linked β-d-xylopyranosyl xylan decorated with branches based on 2D NMR spectra analysis. Lignin was strongly condensed with a decrease of S/G ratio as the severity increased. The yields of the degraded constitutions have a incomplete linear correlation with the treatment severity.

**Conclusions:**

The liquid fractions obtained from hydrothermal treatment were subjected to comprehensive analysis and structural characterization. Results indicated that hydrothermal treatment had a significant influence on the composition and structure of the polysaccharides and lignin in the aqueous phase. The treatment could be adopted to obtain XOS-rich fraction with limited formation of by-products. In addition, the result was expected to further reveal the mechanisms of hydrothermal treatment on rapeseed straw and to facilitate the value-added applications of agricultural residues in the biorefinery industry.

**Electronic supplementary material:**

The online version of this article (doi:10.1186/s13068-016-0552-8) contains supplementary material, which is available to authorized users.

## Background

With the declining of fossil sources, the production of industrial chemicals and fuels from biomass has been studied worldwide. Lignocellulosic biomass, a promising renewable source, is mainly composed of cellulose, lignin, hemicelluloses. Among them, hemicelluloses are polysaccharides which represent a kind of heteropolysaccharide with complex structure including glucan, xylan, mannan, galactan, araban, rhamnan, glucuronic and galacturonic acids in various amounts [1]. They have wide varieties of applications, which can be easily transformed into functional oligosaccharides and further depolymerized into pentose (xylose and arabinose) and hexose (glucose, mannose and galactose) and then converted into bioethanol and high value-added chemicals, such as furfural, 5-hydroxymethylfurfural (HMF) and xylitol [2–5].

Since the components of lignocellulosic biomass are tightly connected through various intermolecular hydrogen bonds and van der Waals forces as well as covalent bonds [6, 7], it is necessary to fractionate them before the conversion process. Many approaches, including chemical, physical and biochemical treatments, as well as a combination of them, have been applied to break the recalcitrance of the lignocelluloses biomass [8–11]. However, from a biorefinery scenario, biomass should be effectively utilized according to physicochemical property of its constituents [12]. In the consideration of economic production, integrated treatment not only enhances production efficiency but also improves productive value. Hydrothermal pretreatment is an environmentally friendly, low cost and effective treatment in the commercial production before downstream biorefinery industry, which is an green innocuous efficient method that utilizes water as the sole solvent under the conditions of high temperature and high pressure in a closed system. The mild pH of the reaction medium avoids problems of equipment corrosion and stages of acid–base handing [13, 14]. The treatment can effectively dissolve and degrade hemicelluloses into sugars with low by-product generation. The degraded sugars present in the liquid fraction mainly consist of high value-added oligomers, which are potentially used for chemicals, food and pharmaceutical production [13, 15]. Hydrolysate is also potential for being directly hydrolyzed by enzyme for the production of bioethanol and represents a resource of bioplatform component for significant industrial use [4, 13, 14, 16]. Additionally, lignin is partially liberated resulting in alteration of the properties of both cellulose and lignin during hydrothermal treatment [9]. Simple operation, low-cost materials of construction, no requirement for chemical addition and high value-added products make the treatment to have great economic advantages [17, 18]. Thus, it is necessary to understand the dissolution mechanism and the products in the liquid phase from the hydrothermal treatment, which would contribute to the biorefinery scenario.

Rapeseed is one of the three main oil-seed crops in the world [19], and over 36 million hectare of rapeseed was cultivated in 2013, which has a great potential to be an energy crop [20]. Rapeseed is traditionally used for the production of vegetable oil and animal fodder. With increasing interest in the renewable energy sources, rapeseed production has increased in recent years as a material for the biodiesel industry [21]. However, most rapeseed straw is directly burned to generate heat and power with low efficiency, which causes great lavish wastes and environmental issues. Therefore, it is necessary to develop methods to transform it into value-added products, enhancing the sustainability of rapeseed production. Previous research on rapeseed straw mainly focused on mineral acid or alkali treatments to improve the yields of fermentation sugars or bioethanol [21–25]. There are several publications about hydrothermal treatment on rapeseed straw, which mainly concentrated on the bioethanol production and the production of xylose as well as glucose [11, 26], but few work concerned on a detailed and systematic evaluation of the hydrolysate produced in the hydrothermal treatment.

The main objective of the present study was to obtain a detailed insight of the constitute changes of rapeseed straw by analysis of the dissolved products (such as oligosaccharides, monosaccharides and water-soluble lignin) and to understand the dissolution and degradation of the lignocellulose constituents during the hydrothermal treatment. The effects of hydrothermal treatment on the degradation products of rapeseed straw were investigated by high-performance anion exchange liquid chromatography (HAPEC), high-performance liquid chromatography (HPLC), thermo-gravimetric and derivative thermo-gravimetric analysis (TGA/DTG) as well as heteronuclear single-quantum coherence (HSQC) in order to reveal the variety of the degraded products from rapeseed straw. In addition, the structural characteristics of soluble products were comprehensively elucidated. These results would provide some valuable information in the commercial exploitation of rapeseed straw for the large-scale production of biobased chemicals in the biorefinery industry.

## Methods

### Materials

Rapeseed straw was harvested in 2015 from a local farm in Shaanxi Province, China. After air-dried, it was manually cut into small sections and ground with a mini-plant grinder. The sample was screened to obtain the fractions with sizes between 20 and 60 meshes. Next, the powder was de-waxed by toluene/ethanol (2:1 v/v) in a Soxhlet extractor for 6 h and destarched with hot water at 80 °C for 2 h [27, 28]. The extractive-free sample was oven-dried at 60 °C for 48 h and stored in a valve bag before use. All chemicals used were of analytical grade.

The extracted rapeseed straw consisted of cellulose 35.01 % (determined as glucan), hemicelluloses 26.78 % (determined as xylan 19.91 %, mannan 2.22 %, galactan 1.18 %, rhamnosan 1.09 %, arabinan 0.94 %, glucuronic acid 1.16 % and galacturonic acid 0.28 %), lignin 13.57 % (Klason lignin 12.70 % and acid-soluble lignin 0.87 %), acetyl groups 3.52 %, ash 2.45 % and moisture 8.17 %. The composition of the solid samples was determined according to the NREL procedure [29, 30].

### Hydrothermal pretreatment

Hydrothermal pretreatment was carried out in a stainless steel autoclave (1000 mL, Parr Instrument Company, Moline, IL, USA) with a mechanic agitation and an electric heater by a PID controller (model 4848). A total of 10.0 g of the extractive-free material was mixed with 200 mL deionized water and incubated at 145, 160, 175, 190 and 205 °C for 15, 30, 60 and 120 min, respectively. The mixture was heated from 30 °C at a speed of approximately 7 °C/min, and the agitation was set at 150 rpm. The reaction temperature was dominated by a ParrCom.exe (1.0.0.9) software. The error range was within 1 °C. Retention time counting was initiated when the temperature of the reaction mixture reached the target value. At the end of each run, the reactor was removed from the heating jacket and cooling water was charged through the serpentine coil. The mixture in the reactor was cooled down to 80 °C in approximately 5 min. The reactor was sealed, and the slurry was agitated until the reactor was cooled to about 40 °C.

After the treatment, the solid substrates were separated by filtration, thoroughly washed with deionized water and dried in an oven at 60 °C until a constant weight. The liquid fractions obtained from filtration were stored to detect the contents of monosaccharides, oligosaccharides and sugar degradation products. The total concentration of oligosaccharides was determined by an indirect method based on quantitative acid hydrolysis of the filtrates in 4 % sulfuric acid at 105 °C for 1 h according to the previous literature [31, 32]. In this procedure, saccharides of DP 2 or higher were considered as oligosaccharides, which is different from the common definition of oligosaccharides (DP, 2–10). The increased concentrations of monosaccharides and organic acids after post-hydrolysis were used to calculate the concentration of oligosaccharides and the amount of aldehyde acid attached on oligosaccharides, respectively. On the other hand, XOS-rich concentrates were obtained by rotary evaporation of the liquids separated at 145, 160, 175, 190 and 205 °C for 60 min as well as at 175 °C for 15, 30, 60 and 120 min and freeze-dried using a lyophilizer (Thermo Modulyo Freeze Drier; Thermo Scientific, Waltham, MA, USA) at −50 °C under vacuum for 48 h, respectively. Accordingly, the solids obtained were noted as L_145–60_, L_160–60_, L_175–60_, L_190–60_, L_205–60_, L_175–15_, L_175–30_ and L_175–120_, respectively.

The severity of hydrothermal treatment was measured in terms of the severity log *R*_0_ and was calculated by the following formula [33], taking account of heating and cooling periods:

$$ \begin{aligned} & \log R_{0} = \log \;\left[ {R_{{0{\text{HEATING}}}} + R_{{0{\text{ISOTHERMAL}}}} + R_{{0{\text{COOLING}}}} } \right] \\ & \quad = \left[ {\log \int_{0}^{{t_{H} }} {\exp \left( {\frac{{T\left( t \right) - T_{\text{REF}} }}{\omega }} \right)} \cdot {\text{d}}t + t \cdot \exp \left( {\frac{{T\left( t \right) - T_{\text{REF}} }}{\omega }} \right) + \int_{{0_{{}} }}^{{t_{C} }} {\exp \left( {\frac{{T\left( t \right) - T_{\text{REF}} }}{\omega }} \right) \cdot {\text{d}}t} } \right] \\ \end{aligned} $$

where *t*_*H*_ (min) is the time needed to achieve the target temperature, *t*_*C*_ (min) is the time needed for the whole heating–cooling period, *t* (min) is the retention time, and *T*(*t*) represents the treatment temperature (°C). Calculations were made based on the values reported in the literature (*ω* and *T*_REF_ are 14.75 and 100 °C, respectively) [33, 34]. According to the above calculation, log *R*_0_ varied in the range of 2.88–5.29 (Table 1), corresponding to the conditions of 145 °C-15 min and 205 °C-120 min, respectively.

**Table 1:** The reaction condition, treatment severity (log *R*_0_), pH of the liquor from the hydrothermal treatment of rapeseed straw

Reaction condition			pH^a^
Temperature (°C)	Retention time (min)	log *R*_0_	
145	15	2.88	4.19
	30	3.08	3.97
	60	3.26	3.78
	120	3.47	3.71
160	15	3.38	3.91
	30	3.51	3.82
	60	3.7	3.74
	120	3.93	3.56
175	15	3.82	3.87
	30	3.99	3.85
	60	4.17	3.74
	120	4.37	3.56
190	15	4.32	3.80
	30	4.44	3.65
	60	4.61	3.47
	120	4.85	3.46
205	15	4.82	3.45
	30	4.92	3.42
	60	5.08	3.42
	120	5.29	3.42

^a^Data represented are mean values of the results obtained from the duplicated determination, and the standard deviation is less than 3 %

### Characterization of the liquid fractions

**Fig. 1:**
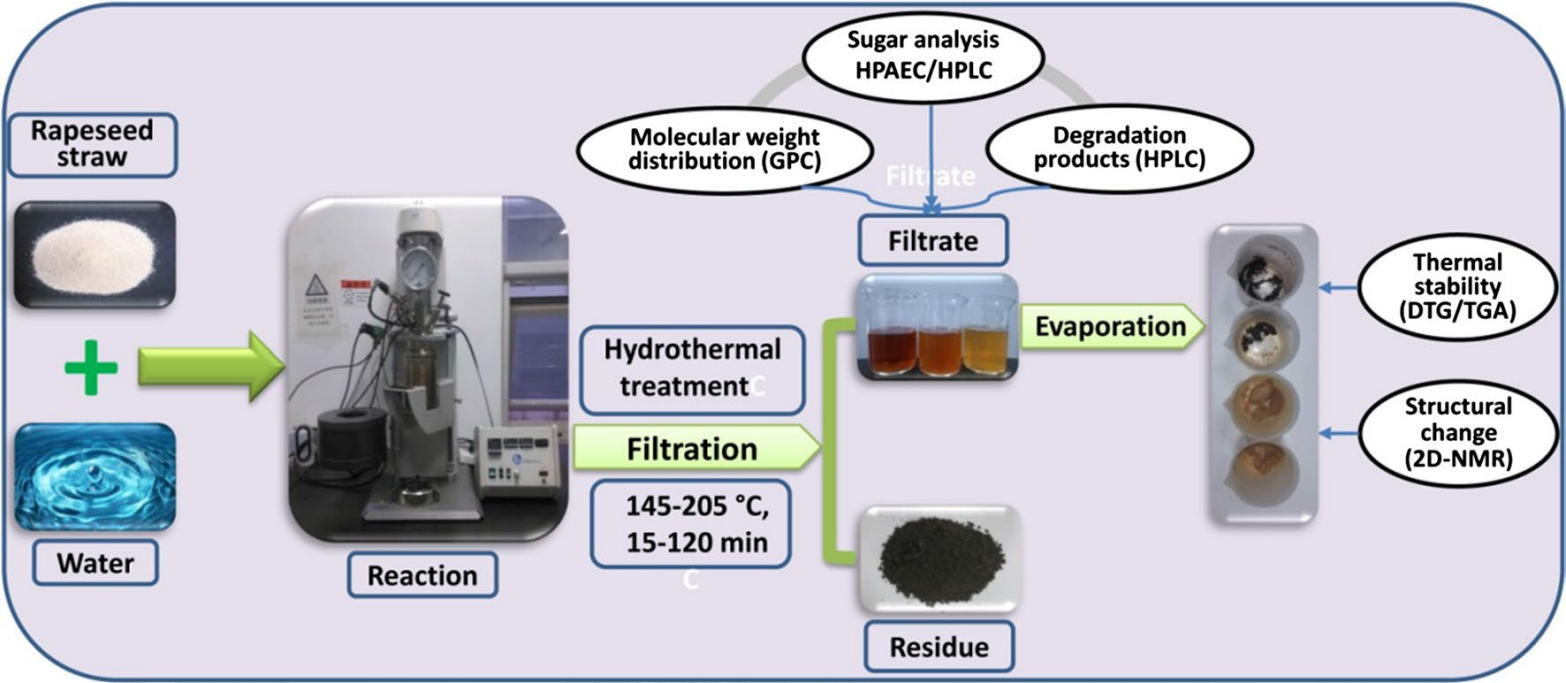
Schematic illustration of the processing of rapeseed straw by hydrothermal treatment

The monosaccharides and XOS (DP, 2-6) of the liquid fractions were determined by a Dionex ICS-3000 HPAEC system with an AS50 autosampler, equipped with a Carbopac PA-20 column (4 × 250 mm, Dionex) for monosaccharides, a Carbopac PA-100 column (4 × 250 mm, Dionex) for XOS (DP, 2-6), according to the method in a previous paper [28]. HPLC (Agilent 1200 series, Agilent Technologies, USA) was used to quantify the degradation products (acetic acid, lactic acid, formic acid, furfural and HMF) in the liquid fractions, according to the method reported in a previous paper with some modifications [35]. All the liquor samples were filtered through 0.22-μm filters before injecting into HPAEC and HPLC systems. Two-dimensional nuclear magnetic resonance (2D-NMR) spectra were obtained on a Bruker AVIII 400 MHz spectrometer. The XOS-rich solids (L_145–60_, L_175–60_, L_205–60_, L_175–15_ and L_175–120_) were dissolved in 0.5 mL DMSO-*d*_*6*_. The number of collected complex points was 1024 for the ^1^H-dimension with a relaxation of 1.5 s. Scan number was 128, and time increments were 256 in ^13^C-dimension. The 1 *J*_C–H_ used was 146 Hz. Prior to Fourier transformation, the data matrixes were zero filled up to 1024 points in the ^13^C-dimension. Thermal stability of the samples was examined using TGA/DTG on a simultaneous thermal analyzer (DTG-60, Shimadzu, Japan). The apparatus was continually flushed with nitrogen. The sample weighed between 3 and 5 mg was heated from room temperature to 700 °C at a heating rate of 10 °C/min. The weight-average (*M*_*w*_) and number-average (*M*_*n*_) molecular weights of the dissolved fractions were determined by GPC using a PL aquagel-OH 50 column (300 × 7.7 mm, Polymer Laboratories Ltd., Church Stretton, Shropshire, UK) with a differential refractive index detector. The data were calibrated with PL pullulan polysaccharide standards. The eluent was 0.02 M NaCl in 0.005 M sodium phosphate buffer (pH 7.5), and the flow rate was 0.5 mL/min according to the method described in a previous paper [36]. All experiments were performed in duplicate, and the results were averaged. Figure 1 shows schematic illustration of the processing of rapeseed straw by the hydrothermal treatment.

## Results and discussion

### Monosaccharide and oligosaccharide analysis of the liquid fractions

The dissolution of hemicelluloses was triggered by hydronium ions generated in situ by autoionization of water and further enhanced by organic acids generated from the degradation of hemicelluloses [37, 38]. The dissolved portions were mainly composed of products degraded from hemicelluloses, such as monosaccharides and oligosaccharides. During this treatment, macromolecules were progressively depolymerized into smaller molecules (oligosaccharides), which formed new intermediates (monosaccharides) for subsequent fragmentation, and then sugar dehydration reactions occurred to form other by-products.

Oligosaccharides degraded from the depolymerization of hemicellulose constituents are mainly composed of xylo-oligosaccharides, which are super-duper prebiotics and have benefits for human health, such as enhancing immunity, promoting *Bifidobacteria* growth, lowing the cholesterol level in serum and preventing diarrhea and constipation [3, 15]. Monosaccharides degraded from oligosaccharides in the liquid fraction mainly consist of pentose (xylose and arabinose) and hexose (glucose, mannose and galactose), which represent a resource of bioplatform component for significant industrial use such as bioethanol, furfural and xylitol [1, 16, 39].

**Fig. 2:**
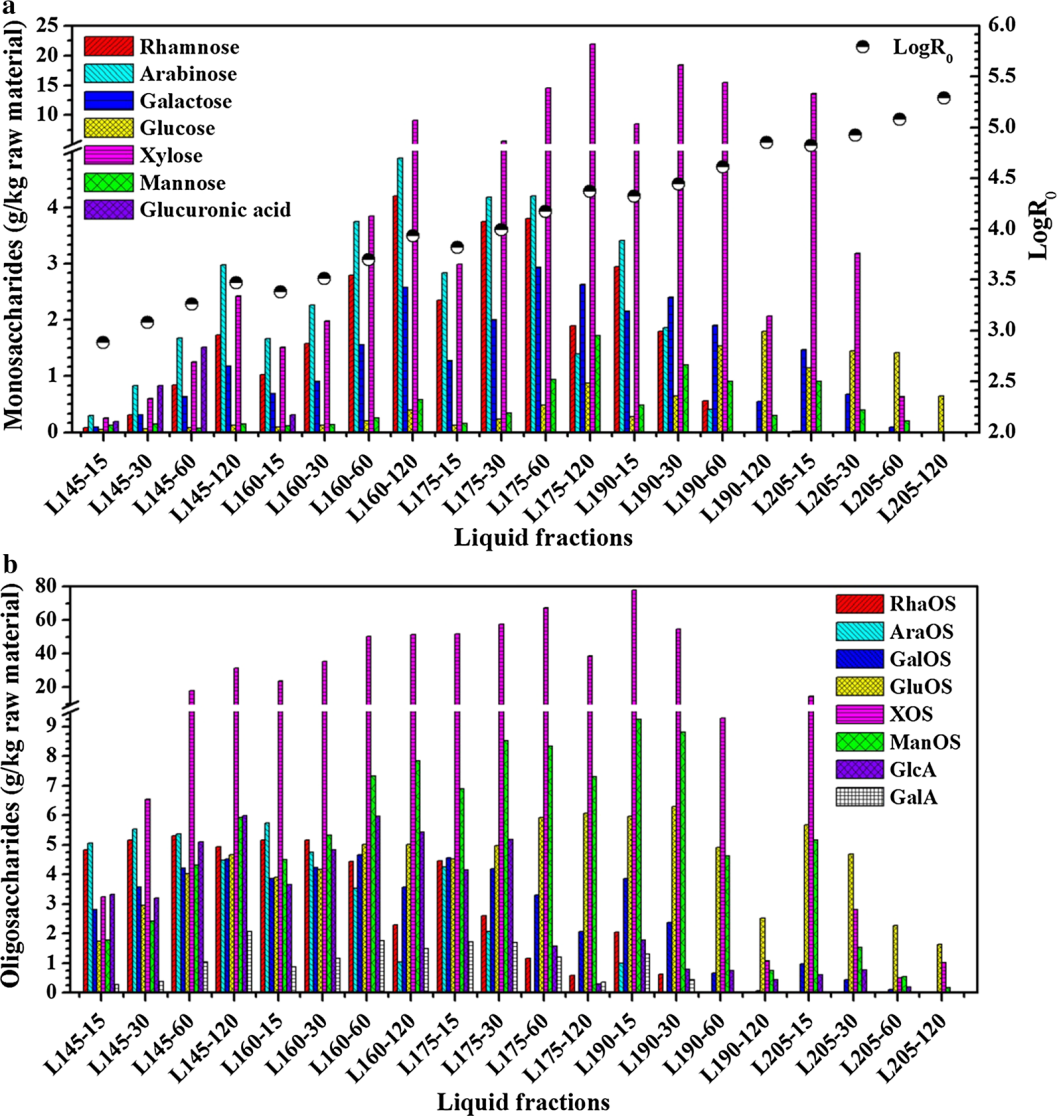
Production of monosaccharides (**a**) and oligosaccharides (**b**) from the hydrothermal treatment of rapeseed straw (*RhaOS* rhamno-oligosaccharides, *AraOS* arabino-oligosaccharides, *GalOS* galacto-oligosaccharides, *GluOS* gluco-oligosaccharides, *XOS* xlyo-oligosaccharides, *ManOS* manno-oligosaccharides, *GlcA* glucuronic acid, *GalA* galacturonic acid). Data represented are mean values of the results obtained from the duplicated determination, and the standard deviation is <3 %

The monosaccharide and oligosaccharide composition of the liquid fractions is shown in Fig. 2a, b. Arabinose had a relatively high concentration as the severity factor was lower than 3.70. Xylose was the main product within the severity range of 3.70–4.92. After severity higher than 4.92, glucose became predominant. Xylo-oligosaccharides (XOS) were the most abundant oligosaccharides in the liquid fraction at the medial severities (3.08–4.82). These results were ascribed to the high content of xylan in the raw material. In addition to xylose and XOS, the amount of monosaccharides in the liquors showed the order: arabinose > rhamnose > galactose > mannose when the severity values were lower than 4.17. Meanwhile, the concentration of other main oligosaccharides showed the order of arabino-oligosaccharides > rhamno-oligosaccharides > galacto-oligosaccharides > manno-oligosaccharides at low severities (log *R*_0_ < 3.08). The maximum concentrations of arabinose [4.88 g/kg raw material (RM)], rhamnose (4.20 g/kg RM), galactose (2.93 g/kg RM) and mannose (1.72 g/kg RM) were achieved at severity values of 3.93, 3.93, 4.17 and 4.37, respectively, while the highest concentrations of arabino-oligosaccharides (5.74 g/kg RM), rhamno-oligosaccharides (5.29 g/kg RM), galacto-oligosaccharides (4.67 g/kg RM) and monosaccharides (9.25 g/kg RM) were obtained at severities of 3.38, 3.26, 3.70 and 4.32, respectively. These results suggested that the extent of the hemicellulose degradation related to the content of the original amount in rapeseed straw. The probable reason was that biomass had a network structure and its constituents interacted with each other by intermolecular forces and covalent bonds. In addition, the high degree of polymerization of hemicellulose constituents or polymer with more branches showed a high resistance to cleave. The concentration of XOS in the liquors increased rapidly at low temperatures and then decreased sharply at high temperatures. The maximum XOS value (77.84 g/kg RM) was obtained at 190 °C for a short retention time of 15 min (log *R*_0_ = 4.32), which was in agreement with the previous report by Carvalheiro et al. [40]. These results illustrated that a proper high temperature at a short retention time may produce a high yield of XOS because a long reaction time and a high temperature accelerated the cleavage of bonds among polysaccharides and the depolymerization of oligosaccharides [28]. Similar to the variation trend of XOS, the yield of xylose in the liquid products firstly increased and then decreased sharply. This was because xylose was produced by the depolymerization of XOS and could be further dehydrated into by-products [5, 16]. The maximum value (21.92 g/kg RM) of xylose was detected at log *R*_0_ = 4.37, indicating that a appropriate long retention time and a low temperature were important for the optimization of the xylose yield. The variation trends of other monosaccharides (arabinose, rhamnose, galactose, mannose) and oligosaccharides (arabino-oligosaccharides, rhamno-oligosaccharides, galacto-oligosaccharides and monosaccharides) were quite similar to those of xylose and xylo-oligosaccharides. Taken as a whole, the highest total oligosaccharide yield (99.94 g/kg RM) was obtained at 190 °C for 15 min, while the maximum total monosaccharide concentrations (30.43 g/kg RM) were achieved at 170 °C for 120 min. At the most severity treatment (log *R*_0_ = 5.29), the concentrations of monosaccharides and oligosaccharides in the filtrates were minimal due to the transformation into by-products. Most of the glucose and gluco-oligosaccharides were produced from the amorphous region of cellulose, and they were gradually released at high severities. Glucose and gluco-oligosaccharides became predominant when the severity was higher than 5.08, similar to the cases in other studies [9, 41].

### Degree of polymerization of the xylo-oligosaccharides

Recently, much work has been focused on the production of xylo-oligosaccharides (especially DP lower than 10), which possess good properties such as promoting the growth of probiotics, favorable effects on the intestinal flora [2, 28, 42]. With the development of membrane separation technique, it is possible to produce xylo-oligosaccharides by hydrothermal treatment in a large scale, which will be investigated in our laboratory during the next 3 years with one industrial company.

**Fig. 3:**
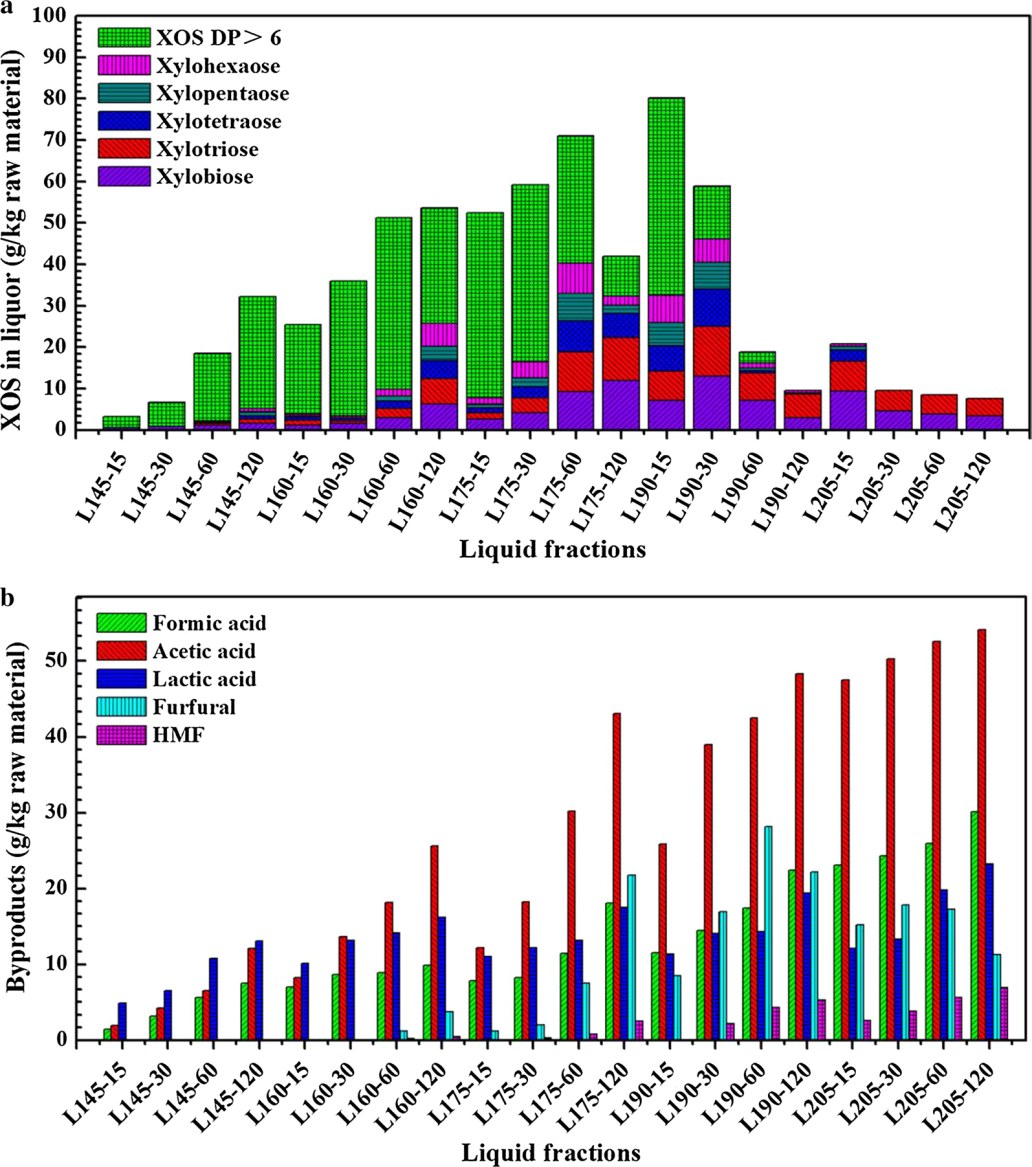
By-products (**a**) and XOS (**b**) from the hydrothermal treatment of rapeseed straw. Data represented are mean values of the results obtained from the duplicated determination, and the standard deviation is <3 %

In this study, the liquid fractions obtained from hydrothermal treatment contained abundant XOS; thus, the content of different degree of polymerization (DP) of XOS was also determined. The total concentration of XOS was measured as monosaccharide after further hydrolyzed. Figure 3a shows the concentration and the DP distribution of XOS, which illustrated that the concentration and DP of XOS were influenced by the treatment temperature and the retention time. The proportion of high-DP XOS was high at mild severities and then decreased with the increase of the treatment severity. Moreover, the proportion of XOS (DP ≤ 6) increased with the increase of the treatment temperature and the retention time because the high-DP XOS was gradually hydrolyzed into low-DP XOS (DP, 2–6) and xylose at the intense severities. A relatively high yield and well-distributed content of XOS were obtained at 175 °C for 60 min as well as 190 °C for 15 min, indicating that a proper high temperature with a short retention time or a low temperature with a long retention time resulted in a high yield and well-distributed content of XOS, which could be oriented toward the production of XOS. The maximum yield of XOS was achieved at 190 °C for 15 min with xylobiose 7.18 (g/kg RM), xylotriose 6.98 (g/kg RM), xylotetraose 6.19 (g/kg RM), xylopentaose 5.59 (g/kg RM), xylohexaose 6.59 (g/kg RM) and high-DP xylo-oligosaccharide (DP > 6) 47.61 (g/kg RM), which corresponded to the xylose analysis of liquid fractions obtained after post-acid hydrolysis treatment. In addition, the maximum yield (46.10 g/kg RM) of low-DP (DP, 2–6) XOS was obtained at 190 °C for 30 min. The high- and low-DP XOS rapidly reduced after a further prolongation of the retention time and increase of the reaction temperature, because xylose was further transformed into other by-products, such as furfural, formic acid, under the harsh treatment conditions.

### By-products analysis of the filtrates

Monosaccharides released from hemicelluloses are degraded into several products, such as furfural, HMF and carboxylic acids (mainly formic and acetic acids), which was reported in previous works [43]. In the present work, the main degraded products of monosaccharides were acetic acid, lactic acid, formic acid as well as a small amount of furfural and HMF (Fig. 3b). The acetic acid obtained by hydrolysis of acetylated part of hemicelluloses rapidly increased with the increase of the severity and showed its highest concentration at log *R*_0_ = 5.29 (205 °C and 120 min). The amount of acetic acid was the highest among all the degradation products when treatment severities were higher than 3.51. The concentration of formic acid in the hydrolysate rapidly increased when the severities were higher than 3.99, and more formic acid was generated from the degradation of furfural [44]. The concentrations of lactic acid, formic acid and acetic acid were well correlated with log *R*_0_ when the temperature was increased at a constant time and the retention time was prolonged at a constant moderate temperature, which were in well agreement with the work of Nitsos et al. [44]. This could be the reason why the pH (Table 1) of the hydrolysate decreased from 4.19 to 3.42 with increasing severity. Furfural gradually increased at a severity value range of 3.70–4.61 and decreased after the severity value higher than 4.61, reaching the highest value of 30.42 (g/kg RM) at the severity factor of 4.61. It was attributed to the formation and degradation reaction of furfural. Initially, the concentration of furfural increased with an increase of treatment severity, but degradation reaction became predominant at the higher treatment severities (4.61–5.29). HMF showed a low concentration due to the low content of nonstructural hexoses in the raw material. A high treatment severity favored to the formation of HMF as compared to furfural. The highest concentration of HMF (6.89 g/kg RM) was obtained at the highest severity of log *R*_0_ = 5.29.

### Molecular weight distribution of the solution fractions

Kinetic studies on hydrothermal treatment of lignocelluloses materials assume that the hemicelluloses are solubilized from the substrates to give high molecular weight of compounds, which are further depolymerized into low molecular oligomers [45, 46]. In addition, the molecular weight distribution of oligosaccharides plays a key role in contributing to their biological properties [32]. Table 2 presents the molecular weight distribution of the liquid fractions after hydrothermal treatment at different temperatures for 60 min and at 175 °C for various times. The results revealed that the weight-average (*M*_*w*_) and number-average (*M*_*n*_) molecular weights of the oligomers declined with the increase of temperature and the prolongation of the retention time, ascribed to the depolymerization of the dissolved polysaccharides. For the curves of molecular weight distributions, a relatively high molecular weight fraction was found at the mild severities. For instance, the *M*_*w*_ of the liquid product obtained at 175 °C for 15 min was 7540 g/mol, while it was 8430 g/mol for the sample obtained at 145 °C for 60 min. In addition, with the increase of the treatment severity, the proportion of high molecular weight decreased and more low molecular weight fraction appeared. This indicated that the high molecular weight oligomers could be obtained at a high temperature for a short retention time. These results were in well agreement with the discussion about the content of different degrees of polymerization of the XOS (DP, 2–6) in the liquid products, similar to the case reported by Vegas et al. [47].

**Table 2:** Weight-average (*M*_*w*_) and number-average (*M*_*n*_) molecular weights as well as polydispersity (*M*_*w*_/*M*_*n*_) of the degraded products in the aqueous phase at different times and temperatures

Reaction conditions		Molecular weight^a^		
Temperature (°C)	Retention time (min)	*M*_*w*_ (g/mol)	*M*_*n*_ (g/mol)	*M*_*w*_/*M*_*n*_
175	15	7540	950	7.94
	30	4670	740	6.31
	60	2230	460	4.85
	120	1440	340	4.24
145	60	8430	1160	7.27
160	60	5720	810	7.06
175	60	2230	460	4.85
190	60	2220	460	4.83
205	60	2130	450	4.73

^a^Data represented are mean values of the results obtained from the duplicated determination, and the standard deviation is <3 %

### Thermal analysis of the dissolved fractions

**Fig. 4:**
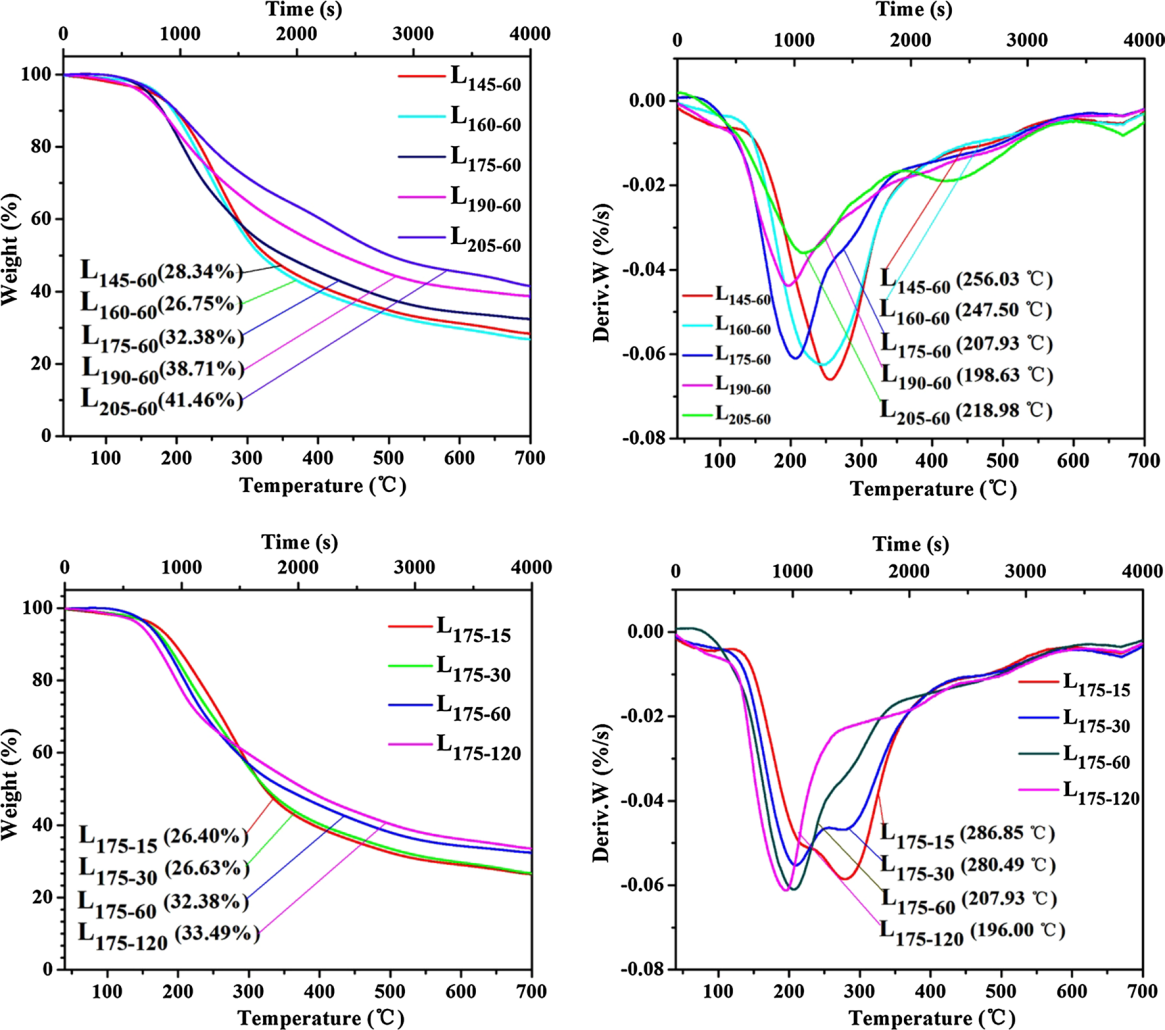
The TGA/DTG analysis of L_145**–**60_, L_160**–**60_, L_175**–**60_, L_190**–**60_, L_205**–**60_, L_175**–**15_, L_175**–**30_ and L_175**–**120_

Thermal properties of polymers are useful for understanding the structure–property relation and application. Figure 4 illustrates TGA/DTG curves of the dissolved fractions L_145–60_, L_160–60_, L_175–60_, L_190–60_, L_205–60_, L_175–15_, L_175–30_ and L_175–120_. In general, the TGA and DTG curves can be divided into three main stages. The initial weight loss of all the products below 150 °C was mainly due to the evaporation of the adsorbed moisture. During the second stage, the severe weight loss mainly occurred at 150–350 °C, which was caused by the XOS degradation, dehydration and decomposition of glycosyl units [48]. The third stage above 400 °C was due to the oxidation and breakdown of the charred residues into gaseous products (CO, CO_2_, CH_4_, CH_3_COOH, HCOOH, etc.) [49]. All the XOS-rich solids showed an early weight loss at 160 and 190 °C, which was due to the thermal decomposition of sugars. The sugar mainly consisted of XOS and branches of hemicellulose, which were easily degraded into volatiles at a relatively low temperature [50]. It was found that the maximum mass loss rate of the samples decreased with the prolongation of the residence time and increase of the treatment temperature due to the generation of small molecules, which was lower than the hemicelluloses extracted by alkali [51]. It should be noted that there were still >25 % solid residues at 700 °C for all samples. This was probably due to salts, water-soluble lignin and ash.

### HSQC analysis of the dissolved fractions

2D ^1^H–^13^C HSQC NMR spectra were used to identify the structural features of the liquid products obtained under the treatment temperatures of 145, 175 and 205 °C for 60 min (L_145–60_, L_175–60_ and L_205–60_) and the retention times of 15, 60 and 120 min at 175 °C (L_175–15_, L_175–60_ and L_175–120_). The provisional peak assignments (see Additional file 1: Table S1) were based on the data reported previously. Common structures found in the liquid fractions are illustrated in Fig. 6b to aid the interpretation of the spectra. The NMR chemical shifts of the hemicelluloses in DMSO-*d*_*6*_ solvent were slightly upfield about 0.1–0.2 ppm for ^1^H and 1–2 ppm for ^13^C from the chemical shifts that obtained in DMSO-*d*_*6*_/pyridine solvents system.

**Fig. 5:**
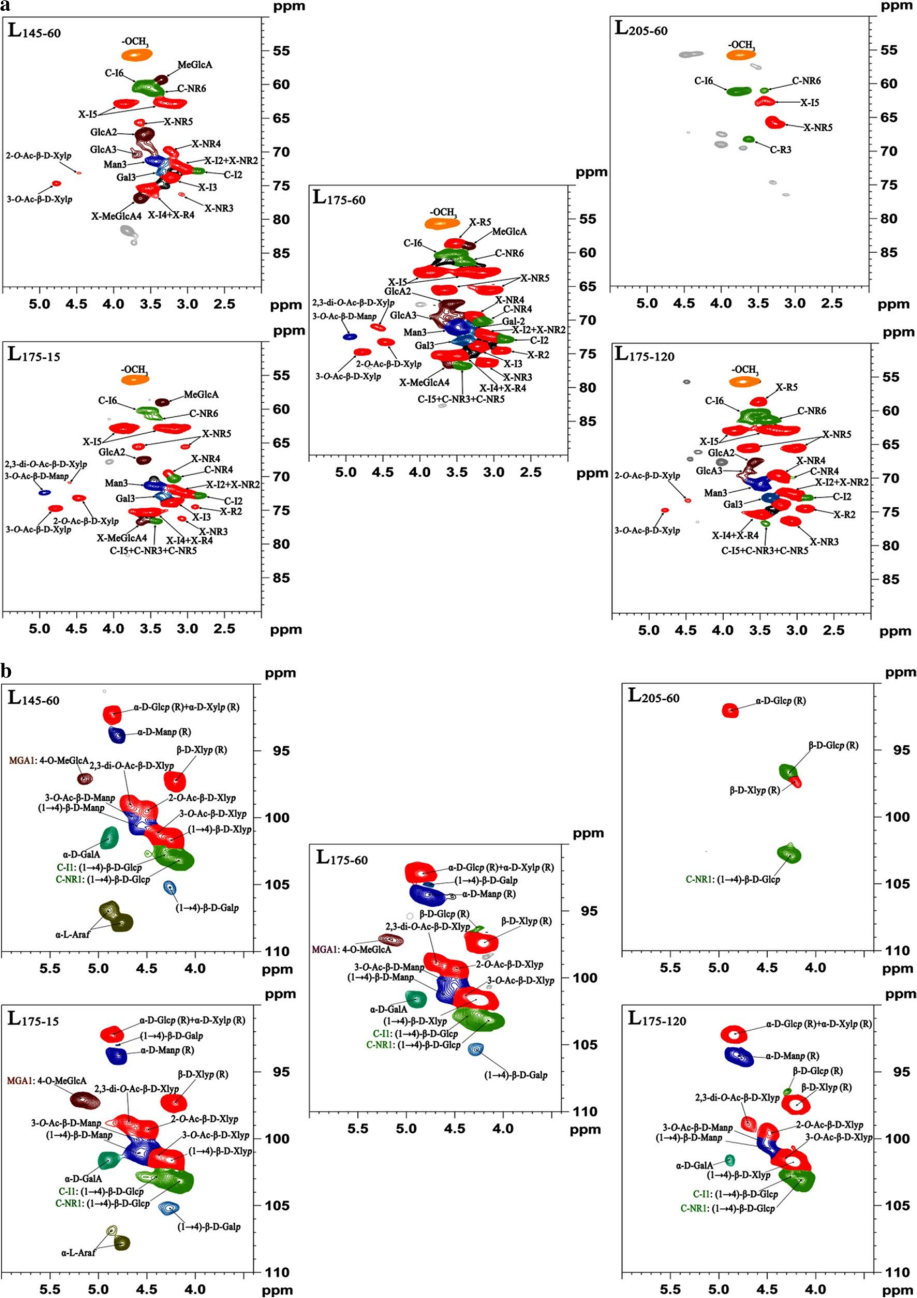
2D-HSQC NMR spectra of the degraded polysaccharides released during the hydrothermal treatment. **a** Aliphatic region and **b** polysaccharides anomerics region

The aliphatic regions of the HSQC spectra of the liquid products before and after hydrothermal treatment are plotted in Fig. 5a. In this region, the methoxyl group (–OCH_3_) was easily identified at 55.68/3.72 ppm. The constituents of hemicellulose cross were predominant in this region. Two strong internal xylan peaks [X-I5 (C5/H5)] located at 62.88/3.21 and 62.88/3.86 ppm, while X-NR5 (C5/H5), two nonreducing end, appeared at 65.50/3.03 and 65.50/3.60 ppm [52]. The residue of xylan backbone X-I2 (C2/H2), X-I3 (C3/H3), X-I4 (C3/H3), X-R2 (C2/H2), X-R5 (C5/H5), X-NR3 (C3/H3) and X-NR4 (C4/H4) showed major peaks at 72.39/3.04, 73.87/3.22, 75.35/3.48, 74.50/2.88, 58.63/3.51, 76.18/3.07 and 69.53/3.20 ppm, but X-I2 and X-I4 shared its chemical shifts with X-NR2 and X-R4, respectively. In addition, 2-acetylated xylan (2-*O*-Ac-β-d-Xylp: C2/H2, 73.13/4.47 ppm) and 3-acetylated xylan (3-*O*-Ac-β-d-Xylp: C2/H2, 74.61/4.75 ppm) were observed in this region [52–54]. However, for the liquid fractions obtained at a constant retention time of 60 min, when the reaction temperature was increased from 145 to 175 °C, the intensity of the cross-peaks of xylan increased. After a further increase of temperature to 205 °C, most peaks vanished other than the stable peak assigned to –OCH_3_, glucan and xylan. Similar phenomenon was observed when the retention time increased from 15 to 120 min at 175 °C.

Mannan, galactan, 4-*O*-methyl-α-d-glucuronic acid (MeGlcA) and α-d-glucuronic acid (GlcA) also showed their peaks at 72.35/4.93 (3-*O*-Ac-β-d-Manp), 71.10/3.50 (Man3), 71.14/3.22 (Gal2), 73.10/3.35 (Gal3), 59.10/3.35 (MeGlcA), 67.60/3.56 (GlcA2) and 69.50/3.62 ppm (GlcA3) [52, 53, 55, 56]. However, when MeGlcA is attached to an *O*-2 position of a xylan residue, the chemical shift of the xylan unit noticeably changes [57]. The X-MeGlcA4 correlation peak appeared at 76.66/3.59 ppm [58]. These peaks only appeared at medium severities between 3.82 and 4.17, which was due to the increase of liberation and degradation of the polysaccharides under the harsh conditions. Celluose (glucan) resonances also appeared in this region. However, due to signal overlap, definitive assignments were difficult to make. The correlations could be identified in this region: the C-I2 (72.76/2.87 ppm), C-I6 (60.20/3.56 ppm), C-NR4 (70.20/3.20 ppm), C-NR6 (61.10/3.39 ppm), while C-I5 shared its peak with C-NR3 and C-NR5 at 76.73/3.43 ppm [52, 54, 59]. The peaks of xylan constituents and glucan became weak from sample L_175–60_ to L_145–60_, which was due to the limitation of the mild hot water treatment. However, once the treatment temperature was further increased to 205 °C, these peaks almost disappeared because of the depolymerization of oligosaccharides and the degradation of monosaccharides. This phenomenon can be reclaimed by the conditions at 175 °C for 15, 60 and 120 min.

Most of the correlations in the region of 90–110/3.5–6.0 ppm belonged to polysaccharide anomerics (Fig. 5b). For the products obtained at 175 °C for 60 min, the anomeric region was assigned to several anomerics, including d-glucan, d-xylan, d-mannan and d-galactan as the major contours. Internal anomerics of the (1 → 4)-linked β-d-glucopyranoside (β-d-Glcp) appeared at 102.80/4.40 ppm and the non-reducing end of (1 → 4)-linked β-d-glucopyranoside (β-d-Glcp) gave a signal at 103.10/4.13 ppm [59]. These two correlation peaks almost appeared in all the spectra except the one obtained at 205 °C for 60 min due to the depolymerization of cellulose and the degradation of glucan. The oligomers revealed the anomeric β-d-mannosyl [(1 → 4)-β-d-Manp)] residues at 100.7/4.60 ppm [60], while the anomeric peak of reducing end of α-d-mannosyl [(1 → 6)-α-d-Manp)] residues appeared at 93.9/4.80 ppm [61]. The anomeric peaks of 3-*O*-Ac-β-d-Manp were observed at 99.90/4.60 ppm [53], which was in agreement with the peak in aliphatic region. The intensity of the correlations assigned to mannan also decreased with the increase of temperature and the prolongation of the retention time, which was due to the depolymerization of mannan. In addition, at the low treatment severity, there are β-d-galactosyl [(1 → 4)-β-d-Galp] internal units and α-d-galacturonic acid groups (α-d-GalA) at 105.32/4.28 and 102.76/4.33 ppm as well as some correlations of α-L-arabinofuranosyl (α-l-Araf) in the area of 106–109/4.7–5.10 ppm [54, 62, 63]. A well-isolated correlation at 97.13/5.15 ppm is assigned to 4-*O*-methyl-α-d-glucuronic acid (4-*O*-MeGlcA) groups [55, 58], which only appeared at a mild treatment severity due to the unstable properties of glucuronic acid. The acetylated xylan structure showed their respective anomeric correlations at 99.31/4.49 ppm (2-*O*-Ac-β-d-Xylp) and 100.97/4.32 ppm (3-*O*-Ac-β-d-Xylp) [53]. The spectra showed that acetyl groups were removed substantially with the increase of the severity. The reducing end of β-d-Xylp was observed at 97.43/4.21 ppm, and the correlation from the α-d-Xylp reducing end was identified at about 92.4/4.85 ppm [55]. In addition, the internal xylan correlation peak [(1 → 4)-β-d-Xylp] from the backbone was observed at 101.61/4.26 ppm [53]. The anomeric correlations for xylan disappeared at the highest severity ascribed to the depolymerization of xylan. The peaks appeared in agreement with the composition analysis of the liquors mentioned above.

**Fig. 6:**
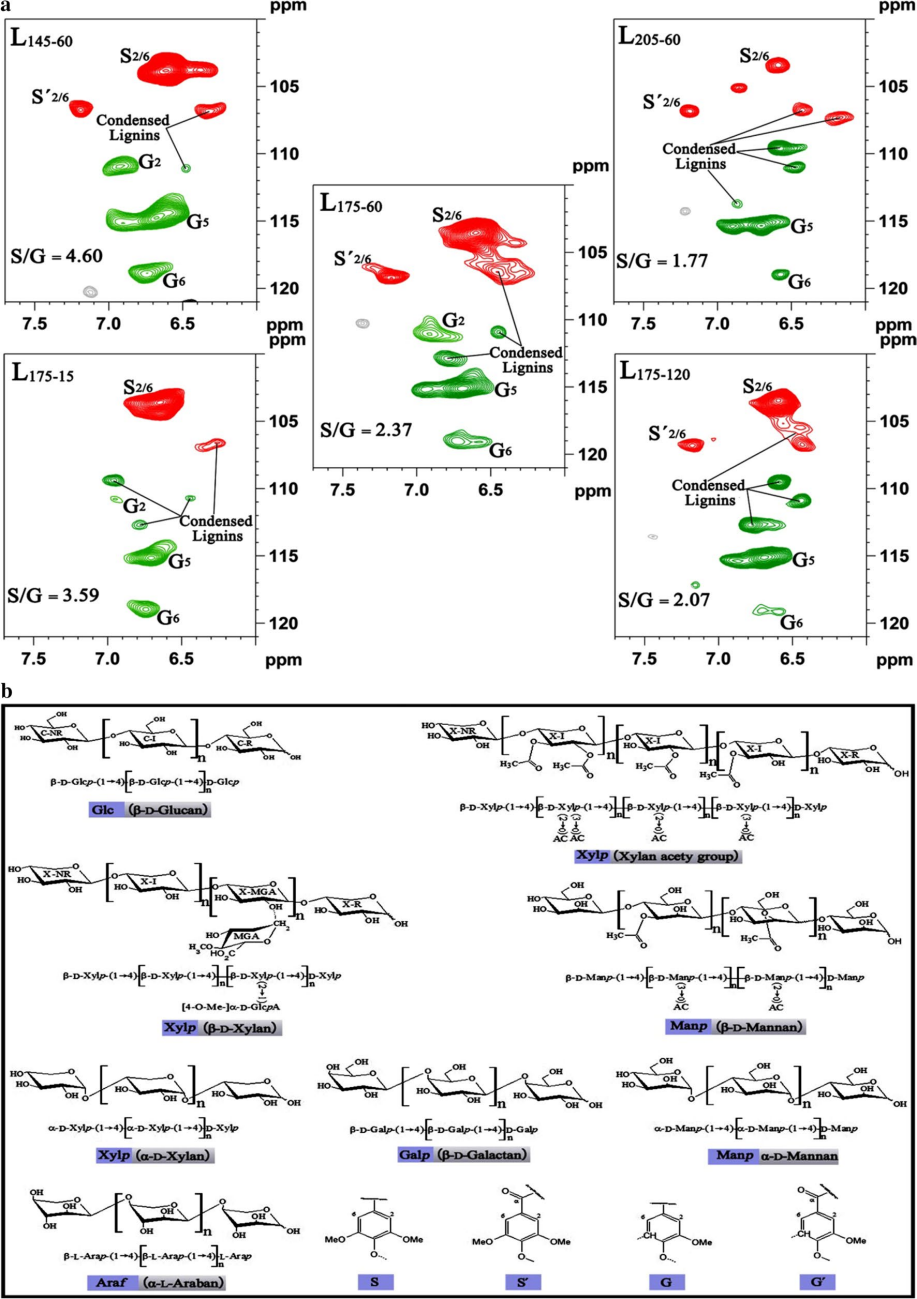
2D-HSQC NMR spectra of the degraded lignin fractions in aromatic region (**a**). Common structures found in the degraded fractions to aid the interpretation of the spectra (**b**, G, guaiacyl units; S, syringyl units; S′, oxidized syringyl units with a C_α_ ketone)

It has been reported that lignin has adverse impact on the process of enzymatic hydrolysis and fermentation by physically impeding the accessibility of enzyme to substrates [64]. It is necessary to pay close attention to the content of lignin in the hydrolysate. The behaviors of water-soluble lignin under the given hydrothermal treatment conditions with increasing severity were elucidated. The aromatic resonances of the aromatic lignin units are depicted in Fig. 6a. The correlations for syringyl (S_2/6_, 103.70/6.60 ppm), oxidized syringyl (S′_2/6_, 106.70/7.21 ppm) and guaiacyl (G_2_, 110.80/6.98 and G_5/6_, 114.70/6.71, 118.90/6.73 ppm) were well resolved [65]. In addition, after hydrothermal treatment, the ratio of syringyl unit to guaiacyl unit of the liquid products decreased with the increase of the treatment severity. The S/G ratio largely decreased from 4.6 in L_145–60_ to 1.8 in L_205–60_ while slightly decreased from 3.6 in L_175–15_ to 2.1 in L_175–120_. This was most likely due to the preferential release of methoxy group in syringyl units than guaiacyl lignin units under the hydrothermal pretreatment. With increasing the treatment temperature from 145 to 205 °C at 60 min or prolonging the retention time from 15 to 120 min at 175 °C, an increase of the content of condensed lignin units was observed. Similarly, the condensation of lignin units has been also observed in a previous study [66]. At the severest condition (log *R*_0_ = 5.08), the signal intensity of syringyl units was sharply decreased, suggesting the degradation of syringyl unit in lignin was the predominant reaction during the hydrothermal treatment at the high severity.

### Correlation between severity factor and yields of the liquid compositions

As shown above, the yields of liquid compositions were incompletely correlated with the treatment severity. A low reaction temperature for a long retention time yielded more monosaccharides and by-products than a high reaction temperature for a short retention time at the closest severity within limited treatment conditions (log *R*_0_ < 4.44). That is why xylose and acid concentrations of L_175**–**15_ were smaller than those of L_175**–**30_ and L_160**–**120_, and the acid concentration of L_190**–**15_ was smaller than those of L_175**–**120_ and L_190**–**30_. However, at the high treatment severities (log *R*_0_ > 4.32), the temperature made more contributions to the treatment efficiency than the retention time on the degradation of monosaccharides, which made the monosaccharide concentrations rapidly decreased except those of L_205**–**15_, because lots of monosaccharides were not degraded at a short retention time. That is why the xylose concentration of L_190**–**120_ was smaller than those of L_205**–**15_ and L_190**–**60_. The results were well in agreement with the previous study [34].

### Process mass balance

**Fig. 7:**
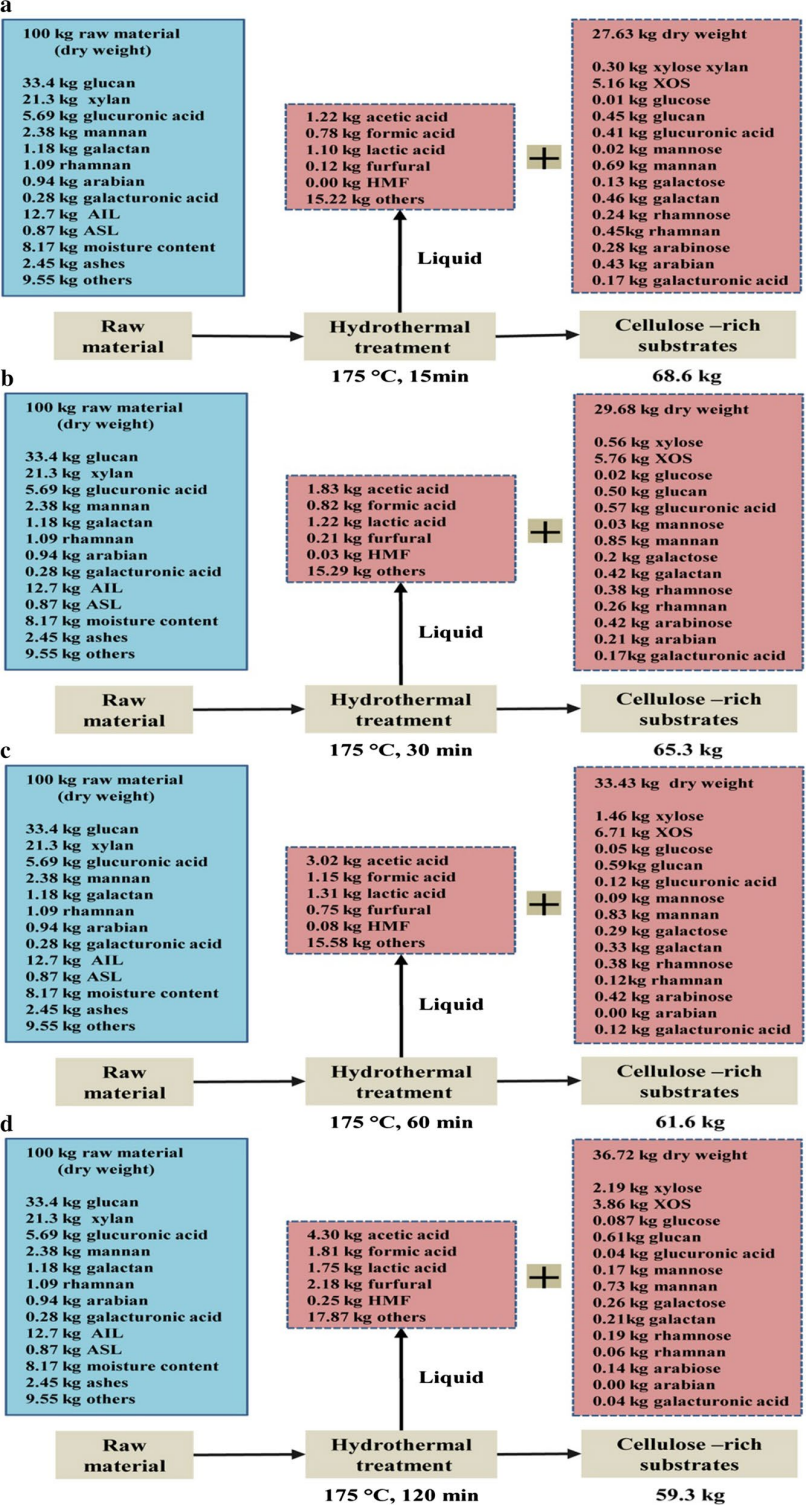
The process mass balance analysis of the products obtained at the reaction temperature of 175 °C for 15, 30, 60 and 120 min based on the raw material. **a** Hydrothermal treatment at 175°C for 15 min, **b** hydrothermal treatment at 175 °C for 30 min, **c** hydrothermal pretreatment at 175 °C for 60 min, and **d** hydrothermal treatment at 175 °C for 120 min

A process mass balance of the hydrothermal pretreatment was developed as shown in Fig. 7. Process yield was normalized to a common basis of 100 kg of dried raw rapeseed straw as the starting material. In the case of the treatment at 175 °C, with increasing the retention time from 15 to 120 min, the yield of the residue decreased from 68.6 to 59.3 kg and the yield of the liquid fraction increased from 27.6 to 36.7 kg. It was found that 5.16, 5.76, 6.71 and 3.86 kg of XOS were obtained when the hydrothermal treatments were performed at 175 °C for 15, 30, 60 and 120 min, respectively. In addition, the degraded products in the liquid fraction were increased with the prolongation of the retention time due to the degradation of the soluble products. These results showed that a relatively high temperature and a short retention time were preferable for the production of XOS.

## Conclusions

Hydrothermal treatment is an green environment-friendly potential valorization process that can be the first step to fractionate hemicelluloses before the downstream refinement. To transform rapeseed straw into value-added products by hydrothermal treatment, a detailed and systematic evaluation of hydrolysate was conducted for the hydrothermal treatment of rapeseed straw at different temperatures and various times. The severity of the hydrothermal treatment had significant influences on the xylan backbone and its side chain constituents. With an increase of treatment severity, the polysaccharides were depolymerized to oligomers and monomers and the corresponded sugars were further degraded. Relatively high temperatures (175–190 °C) and short retention times (15–60 min) were preferred for the production of oligosaccharides, while long retention times (60–120 min) and moderate temperatures (160–175 °C) resulted in high monosaccharide yields. Structural characteristics of the dissolved products were comprehensively elucidated. The 2D NMR gave a visual representation of variation of the oligomers in the hydrolysate during the hydrothermal treatment. The fragmentation of water-soluble lignin occurred during the hydrothermal treatment resulting in its structural changes. The results could in-depthly reveal the mechanisms of hydrothermal treatment on rapeseed straw and facilitate the value-added applications of agricultural residues in the biorefinery industry.

## Additional file

10.1186/s13068-016-0552-8 Assignments of ^13^C-^1^H cross-signals in the HSQC spectra of the liquids fractions obtained during hydrothermal treatment.

## Abbreviations

HMF: 5-hydroxymethylfurfural
2D NMR: two-dimensional nuclear magnetic resonance spectroscopy
HSQC: heteronuclear single-quantum coherence
DMSO: dimethylsulfoxide
HPAEC: high-performance anion exchange chromatography
HPLC: high-performance liquid chromatography
TGA: thermo-gravimetric analysis
DTG: derivative thermo-gravimetric
RM: raw material
XOS: xylo-oligosaccharides
DP: degree of polymerization
*M*_*w*_: mass-average molecular weight
*M*_*n*_: number-average molecular weight
GPC: gel permeation chromatography
