# Supplementary material for: Comprehensive evaluation of the liquid fraction during the hydrothermal treatment of rapeseed straw
Source: Biotechnol Biofuels. 2016 Jul 13;9:142. doi: 10.1186/s13068-016-0552-8 (PMC4944426; doi:10.1186/s13068-016-0552-8)
Supplement: Supplementary file 1 — 10.1186/s13068-016-0552-8 Assignments of 13C-1H cross-signals in the HSQC spectra of the liquids fractions obtained during hydrothermal treatment. [file 13068_2016_552_MOESM1_ESM.doc]

**Additional file 1:**

**Comprehensive evaluation of the liquid fraction during the hydrothermal treatment of rapeseed straw**

Zhi-Wen Wang^1,2^

Email: w_zhi_wen@163.com

Ming-Qiang Zhu^1,2^

Email: [zmqsx@nwsuaf.edu.cn](mailto:zmqsx@nwsuaf.edu.cn)

Ming-Fei Li^2^

Email: [limingfei@bjfu.edu.cn](mailto:limingfei@bjfu.edu.cn)

Jun-Qi Wang^1^

Email: 309280147@qq.com

Qin Wei^1,*^

Email: ma_wei_qin@aliyun.com

Run-Cang Sun^2,*^

Email: rcsun3@bjfu.edu.cn

^1^ College of Forestry, Northwest A&F University, Yangling 712100, China.

^2^ Beijing Key Laboratory of Lignocellulosic Chemistry, Beijing Forestry University, Beijing 100083, China.

*Correspondence : College of Forestry, Northwest A&F University, 712100, Yangling, China. Tel.: +86 29 87082009 (Q. Wei).

Beijing Key Laboratory of Lignocellulosic Chemistry, Beijing Forestry University,

Beijing 100083, China. Tel: +86 10 62336972; fax: +86 10 62336972 (R.C. Sun).

**Table S1. Assignments of ^13^C-^1^H cross-signals in the HSQC spectra of the liquids fractions obtained during hydrothermal treatment**

| **Lable** | ***δ*_C_/*δ*_H_ (ppm)** | **Assignments** |
| --- | --- | --- |
| **–OCH_3_** | 55.68/3.72 | C-H in methoxyls |
| **X-I2** | 72.39/3.04 | (1→4)-β-D-Xyl*p* (C2/H2) of internal xylan |
| **X-I3** | 73.87/3.22 | (1→4)-β-D-Xyl*p* (C3/H3) of internal xylan |
| **X-I4** | 75.35/3.48 | (1→4)-β-D-Xyl*p* (C4/H4) of internal xylan |
| **X-I5** | 62.88/3.21,  62.88/3.86 | (1→4)*-*β-D-Xyl*p* (C5/H5) of internal xylan |
| **X-R2** | 74.50/2.88 | (1→4)-β-D-Xyl*p* (C2/H2) with reducing-end |
| **X-R4** | 75.35/3.48 | (1→4)-β-D-Xyl*p* (C4/H4) with reducing-end |
| **X-R5** | 58.63/3.51 | (1→4)-β-D-Xyl*p* (C5/H5) with reducing-end |
| **X-NR2** | 72.39/3.04 | (1→4)-β-D-Xyl*p* (C2/H2) with non-reducing-end |
| **X-NR3** | 76.18/3.07 | (1→4)-β-D-Xyl*p* (C3/H3) with non-reducing-end |
| **X-NR4** | 69.53/3.20 | (1→4)-β-D-Xyl*p* (C4/H4) with non-reducing-end |
| **X-NR5** | 65.50/3.03, 65.50/3.60 | (1→4)-β-D-Xyl*p* (C5/H5) with non-reducing-end |
| **MeGlcA** | 59.10/3.35 | 4-*O*-methyl-α-D-glucuronic acid (MeGlcA) |
| **GlcA2** | 67.60/3.56 | 4-*O*-methyl-α-D-glucuronic acid (C2/H2) |
| **GlcA3** | 69.50/3.62 | 4-*O*-methyl-α-D-glucuronic acid (C3/H3) |
| **X-MeGlcA4** | 76.66/3.59 | 4-*O*-methyl-α-D-glucuronic acid (C4/H4) linked to xylan at an *O*-2 position |
| **C-I2** | 72.76/2.87 | (1→4)-β-D-Glc*p* (C2/H2) of internal glucan |
| **C-I5** | 76.73/3.43 | (1→4)-β-D-Glc*p* (C5/H5) of internal glucan |
| **C-I6** | 60.20/3.56 | (1→4)-β-D-Glc*p* (C6/H6) of internal glucan |
| **C-NR3** | 76.73/3.43 | (1→4)*-*β-D-Glc*p* (C3/H3) with no-reducing-end glucan |
| **C-NR4** | 70.20/3.20 | (1→4)-β-D-Glc*p* (C4/H4) with no-reducing-end glucan |
| **C-NR5** | 76.73/3.43 | (1→4)*-*β-D-Glc*p* (C3/H3) with no-reducing-end glucan |
| **C-NR6** | 61.10/3.39 | (1→4)-β-D-Glc*p* (C6/H6) with no-reducing-end glucan |
| **Man3** | 71.10/3.50 | (1→4)-β-D-Man*p* (C3/H3) of Manan |
| **Gal2** | 71.14/3.22 | (1→4)-β-D-Gal*p* (C2/H2) of galactan |
| **Gal3** | 73.10/3.35 | (1→4)-β-D-Gal*p* (C3/H3) of galactan |
| **S_2,6_** | 103.70/6.60 | C_2,6_-H_2,6_ in syringyl units (S) |
| **S′_2,6_** | 106.70/7.21 | C_2,6_-H_2,6_ in oxidized (C*_α_*=O) S units (S′) |
| **G_2_** | 110.80/6.98 | C_2_-H_2_ in guaiacyl units (G) |
| **G_5_** | 114.70/6.71 | C_5_-H_5_ in guaiacyl units (G) |
| **G_6_** | 118.90/6.73 | C_6_-H_6_ in guaiacyl units (G) |
